# Correction: Jahan et al. Genome-Wide DNA Methylation and Transcription Analysis Reveal the Potential Epigenetic Mechanism of Heat–Light Stress Response in the Green Macro Algae *Ulva prolifera*. *Int. J. Mol. Sci.* 2025, *26*, 6169

**DOI:** 10.3390/ijms27114735

**Published:** 2026-05-25

**Authors:** Kifat Jahan, Sylvia Kristyanto, Keun-Hyung Choi

**Affiliations:** Department of Earth, Environmental and Space Sciences, Chungnam National University, 99 Daehak-ro, Yusung-gu, Daejeon 34134, Republic of Korea

In the original publication [1], the Acknowledgements section was not included. The following Acknowledgements have now been added. The authors state that the scientific conclusions are unaffected. This correction was approved by the Academic Editor. The original publication has also been updated.

**Acknowledgments:** This work was supported by BK21 FOUR Program by Chungnam National University Research Grant, 2024.
