# Supplementary material for: Genome-Wide DNA Methylation and Transcription Analysis Reveal the Potential Epigenetic Mechanism of Heat–Light Stress Response in the Green Macro Algae Ulva prolifera
Source: Int J Mol Sci. 2025 Jun 26;26(13):6169. doi: 10.3390/ijms26136169 (PMC12249963; doi:10.3390/ijms26136169)
Supplement: Supplementary file 1 [file ijms-26-06169-s001.zip › Table S1.pdf]

Table S1. Key up/down-regulated genes in Glycolysis pathway.

Gene ID		Gene name	Fold Change	Regulation
c796859_g1_i1	phosphoglucomutase	pgm	**-11.665612**	Downregulated
c493770_g1_i1	glucokinase	GCK	**16.457985**	Upregulated
c875191_g1_i1	glucose 6 phosphate	G6PC	**24.341127**	Upregulated
c1047222_g1_i1	aldose 1-epimerase	GALM	**-85.277321**	Downregulated
c1067268_g3_i2	glucose-6-phosphate 1-epimerase	G6PE	**63.944532**	Upregulated
c975494_g1_i1	glucose-6-phosphate isomerase	GPI	**28.615417**	Upregulated
c1086475_g12_i1	fructose-1,6-bisphosphatase I	FBP	**470.337069**	Upregulated
c985305_g1_i1	6-phosphofructokinase	PFK9	**-17.619523**	Downregulated
c1020223_g1_i1	6-phosphofructokinase	PFK10	**15.472352**	Upregulated
c418722_g1_i1	fructose-bisphosphate aldolase, class I	ALDO	**165.161700**	Upregulated
c1076234_g1_i1	triosephosphate isomerase (TIM)	TPI	**63.965658**	Upregulated
c701703_g1_i1	glyceraldehyde-3-phosphate dehydrogenase	gapN	**195.391731**	Upregulated
c1057045_g5_i2	phosphoglycerate kinase	PGK	**165.161700**	Upregulated
c1068909_g9_i1	2,3-bisphosphoglycerate mutase	PGAM, gpmA	**25.794508**	Upregulated
c1080589_g5_i1	2,3-bisphosphoglycerate 3-phosphatase	MINPP1	**25.794508**	Upregulated
c1072175_g1_i2	enolase 1/2/3	ENO1_2_4, eno	**-154.101074**	Downregulated
c939111_g1_i1	phosphoenolpyruvate carboxykinase (GTP)	PCK	**123.617766**	Upregulated
c953163_g1_i1	phosphoenolpyruvate carboxykinase (ATP)	pckA	**223.617766**	Upregulated
c840157_g1_i1	pyruvate kinase	PK	**12.799138**	Upregulated
c106707_g1_i1	pyruvate, orthophosphate dikinase	ppdK	**311.391136**	Upregulated
c408449_g1_i2	pyruvate decarboxylase	PDC	**50.806452**	Upregulated
c611897_g1_i1	dihydrolipoyl dehydrogenase	DLD	**-76.106682**	Downregulated
c210275_g1_i1	aldehyde dehydrogenase (NAD+)	ALDH	**59.983210**	Upregulated
c210275_g1_i1	aldehyde dehydrogenase (NAD(P)+)	ALDH3	**65.918379**	Upregulated
c416781_g1_i1	alcohol dehydrogenase 1/7	ADH1_7	**156.742013**	Upregulated
