# Supplementary material for: Genome-Wide DNA Methylation and Transcription Analysis Reveal the Potential Epigenetic Mechanism of Heat–Light Stress Response in the Green Macro Algae Ulva prolifera
Source: Int J Mol Sci. 2025 Jun 26;26(13):6169. doi: 10.3390/ijms26136169 (PMC12249963; doi:10.3390/ijms26136169)
Supplement: Supplementary file 1 [file ijms-26-06169-s001.zip › Table S2.pdf]

Table S2. Primer list used for qPCR.

Gene ID	Gene Name	Forward	Reverse
c493770_g1_i1	GCK	CGAGTATATGCCAGCGACATT	GTGAGGCATCAACGGATACA
c875191_g1_i1	G6PC	AGTTATCAAACTGGGCCGATTA	GCAAGGAAATCACACGGAATTAG
c1020223_g1_i1	PFK10	GTAGTGGGTAGGGTGAACAATC	AAACCAGAAACCACCTTCCA
c1086475_g12_i1	FBP	CGGGCTTGGTCGACATATT	TGGACAAGGCCTGGATAAAC
c418722_g1_i1	ALDO	GGACAAGGCTCAGAAAGAGAA	CCGGTGGAAACCTGGATAAT
c1057045_g5_i2	PGK	ACAGAGTCGGGTGCATTTAC	TGGACACAAAGCTCACACTATC
c1072175_g1_i2	ENO	GGGTGATCTGGTAGCGAAATAC	CACCCACCAACTGGACTTTAT
c953163_g1_i1	pckA	CAAAGCAGGTCATGCCAATAAA	AGCCCGAAAGGAAGTGATATTC
c840157_g1_i1	PK	GATAATGACCTGGTGGCCTATC	CTGCTTTCCGCAGCAATTC
