# Supplementary figures and images for: Genome-Wide DNA Methylation and Transcription Analysis Reveal the Potential Epigenetic Mechanism of Heat–Light Stress Response in the Green Macro Algae Ulva prolifera
Source: Int J Mol Sci. 2025 Jun 26;26(13):6169. doi: 10.3390/ijms26136169 (PMC12249963; doi:10.3390/ijms26136169)

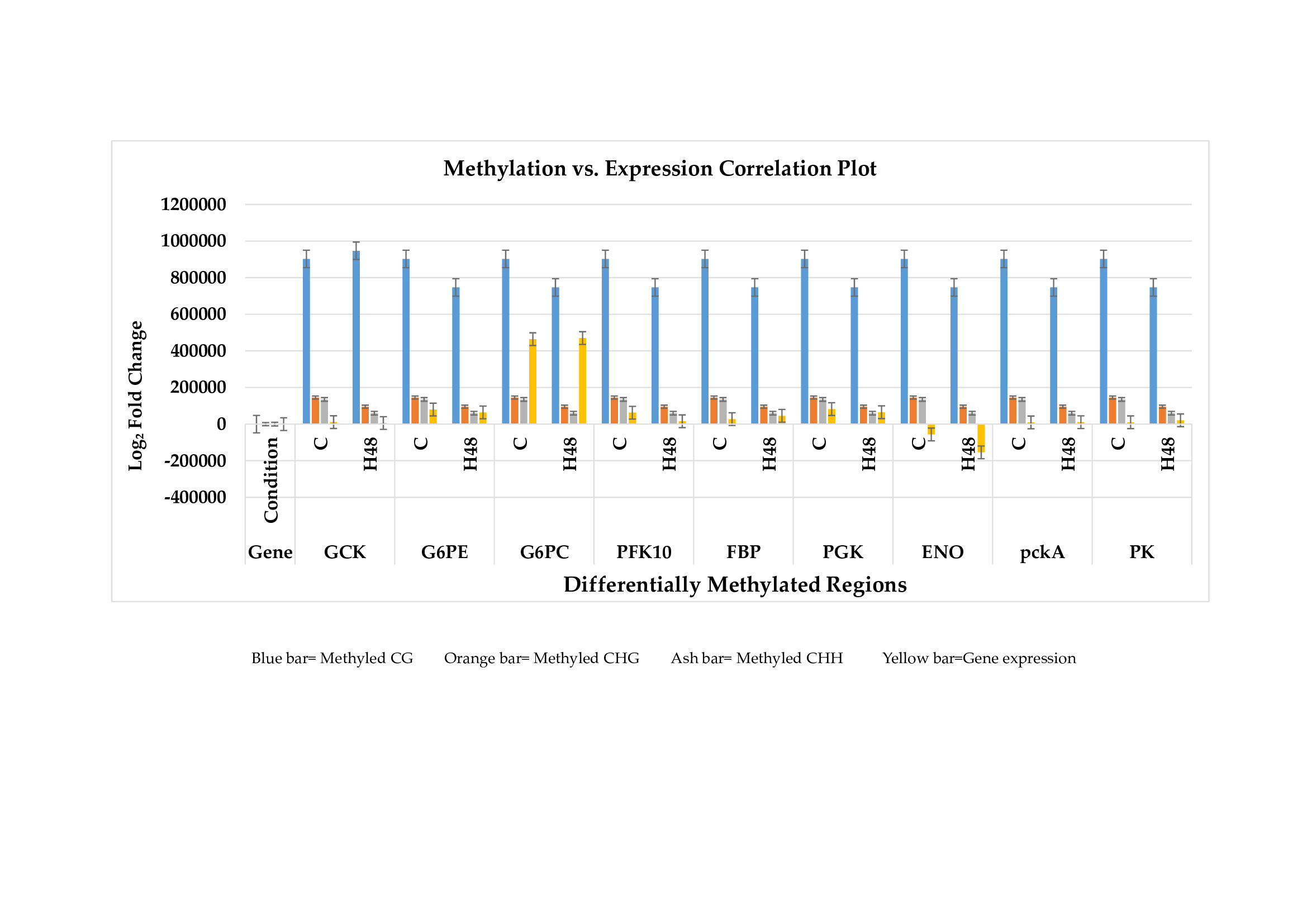

Supplement: Supplementary file 1 [file ijms-26-06169-s001.zip › Figure-S1.png]
